# Carcinogenesis of nasopharyngeal carcinoma: an alternate hypothetical mechanism

**DOI:** 10.1186/s40880-015-0068-9

**Published:** 2016-01-06

**Authors:** Sharon Shuxian Poh, Melvin Lee Kiang Chua, Joseph T. S. Wee

**Affiliations:** Division of Radiation Oncology, National Cancer Centre, 11 Hospital Drive, Singapore, 169610 Singapore; Duke-NUS Graduate Medical School, Singapore, 169857 Singapore

**Keywords:** Nasopharyngeal carcinoma, Carcinogenesis, Epstein–Barr virus, Transformation zone, Toll-like receptor 8 (TLR8), EDAR gene, HLA

## Abstract

Current proposed mechanisms implicate both early and latent Epstein–Barr virus (EBV) infection in the carcinogenic cascade, whereas epidemiological studies have always associated nasopharyngeal carcinoma (NPC) with early childhood EBV infection and with chronic ear, nose, and sinus conditions. Moreover, most patients with NPC present with IgA antibody titers to EBV capsid antigen (VCA-IgA), which can precede actual tumor presentation by several years. If early childhood EBV infection indeed constitutes a key event in NPC carcinogenesis, one would have to explain the inability to detect the virus in normal nasopharyngeal epithelium of patients at a high risk for EBV infection. It is perhaps possible that EBV resides within the salivary glands, instead of the epithelium, during latency. This claim is indirectly supported by observations that the East Asian phenotype shares the characteristics of an increased susceptibility to NPC and immature salivary gland morphogenesis, the latter of which is influenced by the association of salivary gland morphogenesis with an evolutionary variant of the human ectodysplasin receptor gene (*EDAR*), *EDARV370A*. Whether the immature salivary gland represents a more favorable nidus for EBV is uncertain, but in patients with infectious mononucleosis, EBV has been isolated in this anatomical organ. The presence of EBV-induced lymphoepitheliomas in the salivary glands and lungs further addresses the possibility of submucosal spread of the virus. Adding to the fact that the fossa of Rosen Müller contains a transformative zone active only in the first decade of life, one might be tempted to speculate the possibility of an alternative carcinogenic cascade for NPC that is perhaps not dissimilar to the model of human papillomavirus and cervical cancer.

## Introduction

Nasopharyngeal carcinoma (NPC) is the most common cancer originating in the nasopharynx, arising from the squamous mucosal epithelium of the nasopharynx, most often within the fossa of Rosen Müller. Interestingly, NPC is a prevalent cancer in a few specific populations, including natives of South China, Southeast Asia, the Arctic, and the Middle East/North Africa, where the age-standardized rate of NPC incidences can be as high as 21 per 100,000 person-years, compared with the incidence in the rest of the world, which is generally less than 1 per 100,000 person-years [1].

The Epstein–Barr virus (EBV) has been definitively implicated in the pathogenesis of NPC [2]. Other better known factors that contribute to the variation in epidemiological distribution have been dietary preferences rich in nitrosamines, such as preserved foods and salted fish [3, 4], and the inheritance of the human leukocyte antigen (HLA)- and the non-HLA-related genetic susceptibility loci [1, 5, 6].

Wee et al. [7] postulated previously that genetic polymorphisms on Toll-like receptor 8 (*TLR*-*8*) and HLA haplotypes in Southeast/East Asians, as a result of ancient human migration patterns, may have rendered these ethnic groups more susceptible to infection-associated cancers, including NPC. In this paper, we postulate a mechanism for the genetic transmission and subsequent pathogenesis of NPC, building on the ideas of the prior paper, as well as in-depth studies on the epidemiological, genetic, and immunological characteristics of this susceptible patient group.

## Background

### Common genetic susceptibility of Southeast/East Asians to chronic infections

Wee et al. [8] first postulated that all populations at risk of NPC within Asia were traceable by blood linkage to the Bai-Yue ancestral population as a result of the migration histories of the peoples of Southeast Asia.

Furthermore, based on genetic and anthropological evidence, this group might have inherited unique genetic polymorphisms as a result of a genetic bottleneck occurring at the Last Glacial Maximum more than 30,000 years ago, which may explain their susceptibility to chronic infections and, thus, infection-associated cancers such as NPC [8].

### EBV-associated disease patterns

EBV has been implicated in carcinogenesis since the discovery of its implication in Burkitt’s lymphoma in sub-Saharan Africa in the 1960s [9]. The virus has been found to be a potent lymphotropic agent that is capable of transforming B cells in vitro into a state of continuous proliferation called “immortalization.” In recent years, both serological detection of elevated virus titers and molecular techniques permitting detection of latent EBV within tumor cells have reinforced the role of EBV in the pathogenesis of several malignancies [10]. However, EBV-associated conditions have unique patterns of epidemiological predilection.

EBV-associated diseases that are common among East Asians but rare in non-East Asian populations include natural killer (NK)/T cell lymphomas and NPC; in contrast, conditions that are more rarely found in East Asians but are common in the western world include infectious mononucleosis (IM) and its associated conditions—multiple sclerosis (MS) and EBV-related Hodgkin’s lymphoma (HL). It would thus appear that the East Asian phenotype may be vulnerable to some EBV-related conditions but is equally protective against other conditions associated with the same virus.

It has been postulated that early exposure to EBV could contribute to protecting East Asians from IM and MS, which are much more common in western countries [11]. Personal hygiene and genetic transmission have been proposed to result in EBV infection in the early years of life. In any case, it may be logical to infer that early infection predisposes one to NK/T-cell lymphomas and NPC, whereas those who are not exposed to early infection with EBV are more likely to develop IM and MS later in life.

### Genetic susceptibility and chronic EBV infection

Considering the genetic factors and EBV-associated disease patterns, our proposed theory of the mechanism aims to unify these observations and explain the carcinogenic cascade of NPC. We propose that there is a common genetic susceptibility in populations that are prone to NPC as a result of common ancestral origins, which in turn results in a tendency towards EBV chronic infection at an early age. Thereafter, the addition of some environmental insult (such as nitrosamines as previously proposed [4, 5]) results in the malignant transformation of the nasopharyngeal epithelium to NPC.

## Early EBV infection and chronic sino-nasal conditions

### Early EBV infection

Epidemiological studies have always associated NPC with EBV infection in early life [12]. When EBV infection occurs later, perhaps in the teens, it is more likely to be associated with IM and HL.

Considering HL, the incidence pattern of this malignancy follows a conspicuous bimodal age distribution that seemingly varies with the level of socioeconomic development and, thus, environmental risk factors. In the more affluent western hemisphere populations, HL incidence peaks have been observed in younger adults and older adults, but the former is much less apparent in the East Asian population, in which reported incidences of HL in the first four decades of life are extremely low [13].

However, this has changed in recent decades, as illustrated by analyses of data from the population-based Singapore Cancer Registry for the period of 1968–2004 [14], a period during which Singapore had undergone marked improvements in socioeconomic and hygiene standards towards that of affluent western societies. Over time and with the increasing socioeconomic levels, the incidence of HL has been increasing in adolescents and younger adults, with a distinctive incidence peak emerging in these age groups. Currently, cities such as Singapore and Hong Kong have a disease pattern of HL almost similar to that of a Western society. This could be related in part to the possibility that EBV infection is now occurring later in life in these populations. In parallel to this phenomenon is the decline in the incidence of NPC in both Singapore and Hong Kong but not in certain parts of South China, where the socioeconomic status remains low. This finding supports the notion that a delay in EBV infection changes the pattern of disease from NPC to HL.

It is also interesting that an apparent latitude effect exists for MS (which appears to be related to EBV and HL), with this disease affecting mainly those in the temperate countries, except for the Japanese and Greenland Eskimos. However, we interpret this to be more related to “ethnic” predisposition than latitude, and we argue that Japanese and Eskimos, as Mongoloids, are likely more susceptible to early EBV infection than Caucasians. Mongoloid is a phenotype that is associated with the evolutionary variant of the ectodysplasin A receptor (*EDAR*) gene (*EDARV370A*) that rise to straight hair and increased numbers of sebaceous glands. In the same vein, a higher percentage of East Asians (80%) have a unique Toll-like receptor 8 (*TLR8* gene polymorphism) compared with Caucasians and Africans [15].

### Chronic sino-nasal conditions

Historical epidemiological surveys have consistently shown that there appears to be an excess incidence of NPC among individuals who have a history of chronic ear and nose diseases [15–20]. A study from Chang Gung Memorial Hospital, for example, demonstrated that NPC patients had a higher incidence of mucosal sinus abnormalities especially in the posterior ethmoid and sphenoid sinus compared with non-NPC patients, and that males had a higher incidence of sinus abnormalities compared with females [21]. Additionally, other recent papers analysing data from the National Taiwan Insurance database demonstrated that the odds ratio (OR) of prior chronic rhino-sinusitis (CRS) for subjects with NPC is 3.83 [95% confidence interval (CI) 3.23–4.53] compared with controls after adjusting for income, urbanization, geographic location, tobacco use, and alcohol abuse/dependence syndrome [22–24]. Individuals with ≥4 visits for allergic rhinitis per year were also significantly associated with an increased risk of developing NPC [25].

A previous study from the United States Navy further suggested that patients with refractory CRS appeared to suffer from a mild to moderate acquired immune deficiency after surgery that was possibly caused by a chronic EBV infection [26]. Two papers looking for the presence of viruses in specimens obtained from surgery for CRS revealed the presence of EBV, suggesting that EBV may be a cause for the chronic inflammation [27, 28].

What is also intriguing is that CRS in East Asia appears to differ from CRS in the West, with Western CRS having a more eosinophilic infiltration (i.e., allergic), whereas its Asian counterpart is more neutrophilic (i.e., infection-related) [29, 30]. In addition to this well-recognized difference, a study from Singapore demonstrated that a predominant infiltration of lymphocytes, especially CD8^+^ T cells and NK cells, may play a key role in the pathogenesis of nasal polyps and chronic sinusitis [31].

### Pre-malignant EBV serological changes

Most NPC patients present with IgA antibodies to EBV viral capsid antigen (EBV-VCA) and EBV nuclear antigen 1 (EBNA1), and previous studies have shown that such IgA responses often precede tumor presentation by several years [32].

## Mechanism for early and chronic EBV infection

### “3-hit” hypothesis for chronic EBV infection

Wee et al. [7] previously proposed that the stark population differences in the risks for developing NPC could be explained by defects in the subject’s innate immunity and cell-mediated immunity, specifically in *TLR8* and HLA-dependent cell-mediated immunity.

Building on the above information, we now propose a “3-hit” hypothesis for chronic EBV infection as the initial process in the NPC carcinogenesis cascade in these susceptible population groups. It is characterized by the following events in the order indicated: (1) *TLR8* polymorphism leads to faulty innate immunity; (2) early EBV infection occurs during neonatal life; and (3) susceptible HLA leads to faulty cell-mediated immunity.

### “1st hit”: *TLR8* polymorphism

TLRs regulate our innate and adaptive immune response to microbial infections and, more recently, have been shown to play a role in inflammation and the pathogenesis of cancer [33]. Wee et al. [7] previously proposed *TLR8* as a likely candidate for susceptibility to NPC based on an analysis of past genetic and epidemiological research.

First, it has been found that East Asians harbor specific polymorphisms in the *TLR8* gene that are distinct from Caucasians and Africans, which may lead to a functional loss/reduction of this immune mechanism [15]. It is further suggested that these unique *TLR8* polymorphisms are a result of evolutionary selection due to human migration patterns [7]. It is also interesting to note that in the Waldeyer’s ring, *TLR8* expression is highly variable between patients, indicating a further possible difference in susceptibility between patient groups [33].

Another coincidence is the fact that *TLR8* is found on the X-chromosome (Xp22), which could explain a linkage between X-linked recessive polymorphisms in this gene and the 3:1 male to female incidence ratio in NPC. This would be consistent with the proposal of Hu et al. [34] for a South Chinese-specific, recessive NPC gene that is closely linked to the HLA region as a major determinant of NPC associated with this population.

Finally, *TLR8* is the only known mature *TLR* that functions during neonatal life [35]. With increasing age, other classes of *TLRs* are mature and are able to tackle the EBV infection, thus compensating for any negative effects due to an impaired *TLR8* immune response. Collectively, these pieces of evidence implicate *TLR8* in early EBV infection, prior to the progression to a chronic infection.

### “2nd hit”: early neonatal infection with EBV

Previous studies have determined that in regions where NPC is endemic, primary EBV infections are usually acquired at an early age [36]. Serologic surveys have shown that primary EBV infections occur generally under the age of 5 years and, in most cases, occur during infancy in South China, where NPC is most frequent [37–39]. This also appears to be the case in Greenland Eskimos, another population that is at a high risk for NPC and has been postulated to have similar genetic polymorphisms leading to increased EBV chronic infection susceptibility [40].

### “3rd hit”: susceptible HLA

The HLA complex is defined by the genes of the major histocompatibility complex (MHC) located on the chromosome 6 and they code for cell surface proteins that are involved in the antigen presentation for a generation of host immune responses. HLA genes possess a large number of polymorphisms, with more than 1980 unique known alleles and varying allelic frequencies among different racial groups, which result in differences that affect either peptide presentation or cellular interactions with the T-cell receptor and, consequently, inter-individual variation in cell-mediated immunity. With this background, it is entirely plausible to believe that the genetic polymorphisms associated with HLA play a part in inducing susceptibility to EBV-related diseases such as NPC [10, 41].

On this note, a number of studies have reported associations between HLA genes and NPC. A possible association was first identified in Singapore in 1974, when examining an association between the HLA-A2 phenotype and the risk of NPC among Chinese in Singapore [42]. Subsequent studies from high-incidence populations indicated that individuals with A2 and BW46 or with A2–BW46, AW19–B17, and A2–B16 haplotypes are at an increased risk of NPC. The individuals with B17*, B18*, and B35* alleles from high- and mid-incidence populations also have increased frequencies of NPC [43, 44]. Conversely, A*31, B*13, and the associated haplotype A*30–B*13 have been reported to confer protection against NPC [44, 45].

Further insights in this regard can be gained by comparing the predominant HLA gene haplotypes in populations with differing risks of NPC. In the Chaoshanese, a population group in the Guangdong Province of South China with a high incidence of NPC, the HLA alleles B*46, B*38, and B*58 and the related haplotypes A*02–B*46 and A*33–B*58 have been found to be prevalent [45]. These HLA genes have previously been positively associated with NPC susceptibility [6, 45]. In contrast, A*31 and B*13, two alleles that possess highly protective effects on NPC, and the associated haplotype A*30–B*13 were predominantly high in northern Chinese, a population with low incidence of NPC [45].

In a study to investigate East Asian genetic diversity, Di and Sanchez-Mazas [46] found that some HLA alleles observed in southern East Asian populations are virtually unique in Asia. Thus, HLA A*02-07 and B*46-01, which represent the haplotypes with an increased risk of NPC are found mainly in southern East Asian populations and less frequently observed in northern East Asian populations.

Recent genome-wide association studies (GWAS) in susceptible populations also concluded that NPC is associated with HLA genetic variants in the MHC region on the chromosome 6.

In addition, we can examine other EBV-associated diseases for further evidence of HLA-linked genetic susceptibility to EBV chronic infections. Elevated levels of anti-EBV antibodies have been associated with an increased risk of HL. It has been shown that HLA class II variants influence the anti-EBNA-1 IgG titers in a study on a European population, thus reinforcing the role of the same variants in the risk of HL [47]. Separately, Hjalgrim et al. [47] demonstrated that HLA class I-restricted, EBV-specific, cytotoxic T-cell responses and events in the early immune response to IM play critical roles in the pathogenesis of EBV-related HL, whereas Brennan et al. [48] demonstrated that inefficient T-cell control over the proliferation of EBV-positive B cells and malignant, EBV-positive Hodgkin Reed Sternberg cells in individuals with the HLA-A*01 allele bears the same effect. Finally, in a study of pediatric transplant patients, the HLA-A*02 allele expression was predominant in patients with a chronically high EBV viral load [49].

Taken together, these findings support the notion that NPC-prone populations possess variations in HLA gene loci that could contribute to a phenotype of impaired cell-mediated immunity that is prone to chronic infections, in this specific instance, EBV infection.

To conclude, we propose the novel, though untested, “3-hit” model as the scientific basis for a typical East Asian NPC patient to acquire chronic EBV infection:

- A germline *TLR8* polymorphism (80% allele frequency);
- The inheritance of a susceptible HLA gene locus/loci (HLA A*0207 and HLA B*4601 have a 13% and 16% prevalence, respectively, in Hong Kong—the total at risk of susceptible HLA gene would then be approximately 29%) [50]; and
- An infection occurring during neonatal life (assuming a 20% infection rate every 4 months of life [51, 52], or only 5% per month); with increasing age, other TLRs will mature and reduce the likelihood of EBV infection.

Extrapolating from these numbers, the crude estimate of the “incidence” of individuals fulfilling all three criteria would be 1.2% (0.8 × 0.3 × 0.05 = 0.012 or approximately 1 per 100 persons), which is somewhat in accordance with the reported incidences of NPC in Hong Kong [53].

Four studies have examined the presence of EBV in “normal” Chinese adults. Chan et al. [54] and Sam et al. [55] failed to detect EBV in 23 “normal” nasopharyngeal biopsies each. In contrast, Tsai et al. [56] found EBV by polymerase chain reaction in 7 of 61 patients without neoplasia, and Huang et al. [57] found that only 2 of the 202 healthy subjects who had elevated antibody levels were DNase-positive. Thus, of the 309 total “normal” nasopharyngeal biopsies, 9 were found to have EBV, or approximately 3 per 100 “normal” subjects, as per our estimates.

## Salivary gland cell: the epithelial cell harboring EBV

### Evidence for NPC’s association with salivary gland cells

NPC is widely considered to be squamous in origin, although Li et al. [58] proposed that at least one variant of NPC might arise from the basal cells of respiratory epithelium. That said, an intriguing fact of EBV-associated NPC is the inability to detect EBV in the nasopharyngeal epithelium of high-risk individuals; however, the virus is persistently detected, even in the early stages of malignant transformation. One thus wonders whether EBV resides in other anatomical sites in the latent stages following acute infection. A possible site could be the minor salivary glands in the nasopharynx or the adjacent oropharynx. The occurrence of “Eskimoma”, a lymphoepithelial carcinoma of the parotid gland that is characterized by raised EBV serology and a similar histology to NPC, raises this possibility [59]. Lymphoepithelioma-like carcinoma of the lung is another example, and is indistinguishable from undifferentiated NPC [60]. In IM, where primary transmission is through the saliva, EBV is detectable in non-cancer lesions of the salivary glands [61–63], again supporting the notion of the salivary gland cell as a possible nidus for EBV in the latent phase.

Although NPC is considered histologically to be of squamous origin, several pathologists have questioned this. Li et al. [58] and Lin et al. [64] proposed that at least one variant of NPC might arise from the basal cells of the respiratory epithelium.

NPC is also known to spread sub-mucosally. Sham et al. [65] demonstrated that approximately 14% of patients have the sub-mucosal growth pattern in NPC. Occult microscopic extension of a tumor that was not detected on endoscopy was found in a further 50% of patients.

Another provocative suggestion in this regard relates to the prevalence of *EDARV370A* among East Asians. *EDAR* is a cell-surface receptor for ectodysplasin A and plays a pivotal role in the development of ectodermal tissue. Jaskoll et al. [66] demonstrated that the *EDAR* gene signaling is essential for embryonic submandibular salivary gland development, whereas Kallapravit et al. [67] demonstrated that the histological structures of the sebaceous glands in adult Siamese seemed to be more juvenile than those of white and black Americans. It is thus possible that that the abundance of immaturely formed minor salivary glands found in the epithelium of East Asians provides a more conducive nidus for EBV.

### Mouse model

In a mouse model, Ptaschinski et al. [68] demonstrated that infection of the neonatal mouse with murine gammaherpesvirus-68 (γHV-68) results in an enhanced viral persistence in the lungs and an absence of IM syndrome. They observed that the persistence of the herpes virus is age-dependent, as is the development of IM-like syndrome. *TLR8* is non-functional in mice because it lacks five amino acids [69], which may explain why γHV-68 is found in the lungs of mice when they are infected as pups.

## Transformation zone in the fossa of Rosen Müller

### Histology of the fossa of Rosen Müller

The submucosal glands of the nasopharynx have the histological structure of a salivary gland, being composed of mucous and serous acini, as well as mucous acini with serous demilunes [70].

It has been demonstrated that in the nasopharynx, there is a transitional zone between the ciliated columnar respiratory and the stratified squamous epithelium [71]. This intermediate epithelium showed gradations ranging from stratified low-columnar through stratified cuboidal to stratified squamous type [72]. However, the presence of this transformational zone is only active during the period of fetal development to the first 10 years of life [73]; thus, any changes to the epithelium induced by viral insults must occur early in life, which would coincide with the period when *TLR8* is the only active regulator of the host immune response.

The intermediate epithelium appears to be particularly susceptible to oncogenic stimuli. It is therefore no coincidence that the areas of the nasopharynx that are the primary sites of carcinomas are also where the intermediate epithelial cells are found in their greatest numbers [74].

### Carcinogenic cascade

The subsequent development of NPC within the nasopharynx may then follow a “cervical cancer model.” Infection with the human papillomavirus (HPV) has been established as the primary process for the development of cervical cancers. At low passage numbers, HPV-immortalized cells are non-tumorigenic. They can undergo malignant progression after extended growth and exposure to carcinogens or when additional oncogenes are expressed. Similarly, the progression of high-risk HPV-positive cervical lesions is a protracted process that occurs at a low frequency and requires the acquisition of host genetic and epigenetic mutations [75]. These changes exist primarily at the transformation zone, a region where metaplastic squamous cells are detected in otherwise columnar epithelial-lined endo-cervical glands. Women usually contract HPV between their late teenage years and early 30s, when the transformation zone is the largest, but there is a long latency to the onset of cervical cancer (peak incidence at 45 years of age) [76–78].

We postulate that NPC carcinogenesis follows a similar model. Neonatal transmission of EBV infects the nasopharyngeal epithelium with resultant latency in the basal epithelium (salivary gland cells) during the developmental period of the transformational zone. Entry of the virus into the epithelial cells may perhaps be mediated in this case by integrin β6 [78]. Exposure to subsequent carcinogenic insults triggers the carcinogenic cascade that takes years for the eventual malignant transformation to NPC.

## Testing the hypothesis

Methods that can be used to investigate this hypothesis include the following:

1. Histology and pathology of the fossa of Rosen Müller
  • Study the temporal changes that occurred in the fossa of Rosen Müller (1) from autopsy studies of patients in highly endemic regions who died of other causes; (2) from biopsies taken during surgery for other non-NPC otolaryngological conditions in patients in highly endemic NPC regions; and (3) from biopsies from subjects in NPC screening studies who manifested different combinations of EBV serological and EBV DNA titers.
2. Mouse models
  • Compare the effects of γHV-68 infection in the pups of wild type (BALB/c) [68], *EDAR370A* transgenic mice [79], and *TLR8* transgenic mice [80] (the mouse and human nasopharynx are comparatively similar in terms of the epithelium, the presence of an intermediate zone, and the presence of sero-mucinous glands in the submucosa [81]. *TLR8* is non-functional in mice because it lacks five amino acids [71], which might explain why γHV-68 is found in the lungs of mice when they are infected as pups).
  • Expose γHV-68-infected mice (wild-type, *EDAR370A*, and *TLR8* transgenic) to chronic chemical carcinogens.
  • Expose γHV-68-infected rats (infected during neonatal period) to chronic chemical carcinogens [82, 83].
3. HLA haplotype
  • Determine the HLA haplotype of a reasonable size cohort of NPC patients.
4. Large animal models
  • Use lymphocryptovirus (LCV) and TLR8 antagonists to mimic early neonatal EBV infection in neonatal rhesus monkeys.
  • Test neonatal EBV vaccination strategies.
5. EBV vaccination
  • Perform large-scale, population-based, case–control study of EBV vaccine in neonates, with long-term follow-up for NPC patients (currently, the only clinically available prophylactic EBV vaccine is gp350, which has only demonstrated efficacy for the reduction of the rate of IM, but not virus infection).

## Conclusions

The mechanism and hypothesis set forth in this paper offer a convenient explanation for many of the enigmatic characteristics of this peculiar cancer, addressing the following points:

1. The predominant Southeast Asian distribution: the *TLR8* polymorphism is an East Asian signature, and the HLA haplotypes associated with it are South Chinese and Southeast Asian haplotypes;
2. The apparent increased susceptibility in certain families and the presence of multiple cases of NPC in some extended families: families with index cases would already have the appropriate *TLR8* and HLA present and are most likely to be subjected to the same practices and environmental insults, which would predispose them to “chronic EBV infection” and carcinogenesis;
3. The apparent absence of EBV in “normal” nasopharyngeal biopsies: our hypothesis proposes that the virus will only be found when all three factors in our “3-hit” hypothesis are present or, alternatively, when other anatomical sites are the potential nidus for EBV in the latent phase, thus explaining the lack of EBV in the “normal” individuals sampled;
4. Circumstantial evidence for “chronic EBV infection” occurring early in life;
5. The unique role of the East Asian phenotype and EBV-related conditions; and
6. The decreasing incidence of NPC in HK and Singapore, and the corresponding increase in incidence in early HL in the same cities, as explained by the acquiring of the chronic EBV infection later in life as a result of improved socioeconomic factors.

In addition, the HPV “cervical cancer” model provides a mechanism that ties in the factors noted above to the proposed carcinogenesis cascade, thus completing the picture further.

Some suggested means to test this hypothesis include in-depth temporal histological and pathologic studies of the fossa of Rosen Müller; the use of mouse (and rat) models—exposing them to γHV-68 followed by chemical carcinogens; the determination of the HLA haplotype of NPC patients, and using large animal models to test a neonatal EBV vaccination strategy. Vaccination strategies may ultimately reduce the incidence of this cancer in endemic areas.
